# Mechanical Properties of Biodegradable Fibers and Fibrous Mats: A Comprehensive Review

**DOI:** 10.3390/molecules30153276

**Published:** 2025-08-05

**Authors:** Ehsan Niknejad, Reza Jafari, Naser Valipour Motlagh

**Affiliations:** Department of Applied Science, University of Quebec at Chicoutimi (UQAC), 555, Boul. de l’Université, Chicoutimi, QC G7H 2B1, Canada; eniknejad@etu.uqac.ca (E.N.);

**Keywords:** biodegradable polymers, mechanical properties, micro/nanofibers, fibrous mats, tensile tests, electrospinning, centrifugal spinning, synthetic and natural polymers

## Abstract

The growing demand for sustainable materials has led to increased interest in biodegradable polymer fibers and nonwoven mats due to their eco-friendly characteristics and potential to reduce plastic pollution. This review highlights how mechanical properties influence the performance and suitability of biodegradable polymer fibers across diverse applications. This covers synthetic polymers such as polylactic acid (PLA), polyhydroxyalkanoates (PHAs), polycaprolactone (PCL), polyglycolic acid (PGA), and polyvinyl alcohol (PVA), as well as natural polymers including chitosan, collagen, cellulose, alginate, silk fibroin, and starch-based polymers. A range of fiber production methods is discussed, including electrospinning, centrifugal spinning, spunbonding, melt blowing, melt spinning, and wet spinning, with attention to how each technique influences tensile strength, elongation, and modulus. The review also addresses advances in composite fibers, nanoparticle incorporation, crosslinking methods, and post-processing strategies that improve mechanical behavior. In addition, mechanical testing techniques such as tensile test machine, atomic force microscopy, and dynamic mechanical analysis are examined to show how fabrication parameters influence fiber performance. This review examines the mechanical performance of biodegradable polymer fibers and fibrous mats, emphasizing their potential as sustainable alternatives to conventional materials in applications such as tissue engineering, drug delivery, medical implants, wound dressings, packaging, and filtration.

## 1. Introduction

**Figure 1:**
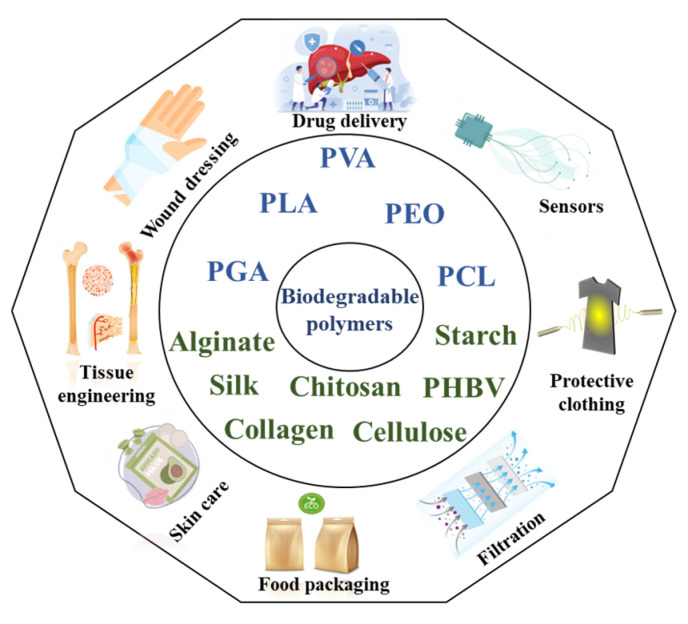
Types of biodegradable polymers and their applications.

Biodegradable polymer fibers and nonwoven mats are increasingly recognized for use in applications such as tissue engineering scaffolds, drug delivery systems, filters, and biosensors because of their high surface-area-to-volume ratio, porosity, and ease of functionalization [1,2,3]. Polymers like polyvinyl alcohol (PVA), polylactic acid (PLA), polyhydroxyalkanoate (PHAs), polycaprolactone (PCL), polyglycolic acid (PGA), and cellulose have gained considerable interest for their use in fiber production because of their sustainable nature [4,5,6,7]. These biodegradable fibers are used in such contexts as agriculture, healthcare, and packaging (Figure 1), help reduce plastic pollution, and are deemed more environmentally friendly than alternatives [8,9,10]. Various production methods for biodegradable polymer fibers and nonwoven mats offer distinct advantages. Electrospinning, a common technique, produces ultrafine fibers with diameters ranging from nanometers to micrometers. Electrospun nanofibers could possess exceptional mechanical properties, high specific surface area, and fine porosity, making them ideal for tissue engineering and drug delivery applications [11]. Although electrospinning is prevalent for polymer nanofiber production, methods like melt blowing, centrifugal spinning, and wet spinning also offer benefits for large-scale manufacturing, cost-effective commercialization, and control of processing [12]. The tensile strength, flexibility, and durability of these materials are vital for demanding applications such as medical implants and protective clothing. Thus, understanding the mechanical behavior of mats and fibers is essential for selecting suitable methods and optimizing process design. Recent advancements include developing biodegradable fibers through copolymerization, crosslinkers, and pre- and post-treatment techniques to enhance mechanical properties [13,14,15,16].

The mechanical properties of fibers can be influenced by various parameters, including (a) the inherent physical properties of the polymer, (b) mat density, (c) average fiber diameter, (d) fiber morphology, and (e) fiber orientation in the mat [17,18]. This review aims to provide a comprehensive overview of the mechanical properties of biodegradable fibers and nonwoven mats, focusing on different production methods. Achieving optimal performance in structure, morphology, strength, and cost-effectiveness requires considering alternative fiber production methods beyond electrospinning. To our knowledge, similar review papers in this area are lacking. This paper gathers and analyzes mechanical reports from both older and recent studies, emphasizing key parameters such as tensile strength, tensile modulus, and elongation. We also discuss specific processing parameters that affect fiber morphology and mechanical properties. Comparing mechanical property data from different researchers is challenging because of variations in production methods, measurement units, and testing conditions. Therefore, we focus on identifying gaps and areas for future improvement in this field.

## 2. Fiber Manufacturing Methods

This section presents various methods for producing fibers and mats, highlighting their specifications, benefits, and drawbacks. The discussion provides insights into production processes to help guide the selection of the most suitable method on the basis of polymer types, production speed, and fiber quality.

### 2.1. Electrospinning

**Figure 2:**
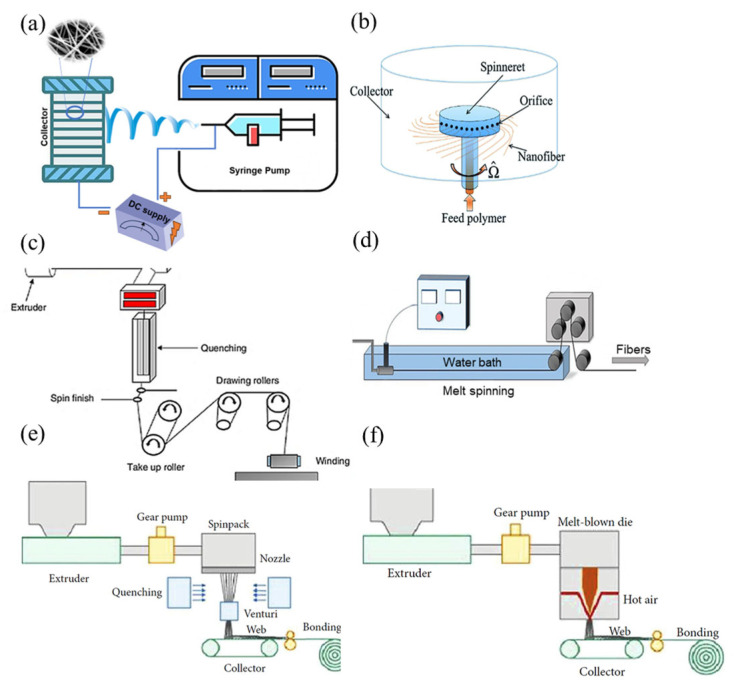
Schematics of (**a**) electrospinning [19], (**b**) centrifugal spinning [22] (reprinted with permission), (**c**) melt spinning [23] (reprinted with permission), (**d**) wet spinning [24] (reprinted with permission), (**e**) spunbonding process, and (**f**) melt blowing [25] (reprinted with permission).

Electrospinning is a versatile and effective method for producing ultrafine fibers with diameters ranging from nanometers to micrometers. The process applies a high voltage to a polymer solution, ejecting it through a thin needle. The charged polymer jet elongates because of electrostatic repulsion until it solidifies and deposits onto a grounded collector, forming a nonwoven fabric of fibers (Figure 2a) [19]. The key parameters affecting electrospinning include solution properties (viscosity, conductivity, surface tension, volatility), process conditions (electric field strength, distance from tip to collector, feed rate, needle tip diameter), and ambient factors (temperature, humidity) [20]. By adjusting these parameters, one can control fiber properties such as morphology, size, and arrangement for various applications [21].

Despite its advantages, including a high surface-to-volume ratio, porosity, and the ability to create composite fibers, electrospinning has drawbacks. It is sensitive to parameter variations, requiring precise control to achieve consistent quality [26,27]. Additionally, its production rate is lower than that of other industrial processes, which limits commercial scalability [28]. Another disadvantage is the need for high voltage, which raises operational safety concerns. However, ongoing research continues to develop solutions that address these limitations and expand the applicability of electrospun materials [29].

### 2.2. Centrifugal Spinning

Centrifugal spinning is a fiber fabrication technique that uses centrifugal forces to produce microfibers and nanofibers from polymer solutions or melts. In this method, illustrated in Figure 2b, the polymer fluid is loaded into a rotating spinning head with multiple nozzles arranged radially [14]. The rotation generates high centrifugal force, ejecting polymer jets through the nozzles, which are then elongated by air drag and deposited on a collector. The key parameters in this process include rotational speed, solution viscosity and concentration, nozzle geometry and size, spinning distance, and solution feed rate [15]. By adjusting these parameters, fibers with tailored morphologies and dimensions can be produced. Compared to other fiber fabrication methods, centrifugal spinning offers several advantages, such as high production rates, cost-effectiveness, versatility in usable polymer types, and ease of process control and scalability. However, it has limitations, including a relatively broad fiber diameter distribution and challenges in collecting nanofibers because of high-speed rotation [15,16].

### 2.3. Melt Spinning

Melt spinning is a fiber production process in which molten polymer is extruded through a tiny nozzle and stretched during cooling to form the fiber. As shown in Figure 2c, this process includes a screw extruder barrel, a metering pump, a die head, an air-cooling system, a spin finish, and a winding system [17]. Polymers are fed through the hopper into the extruder, where they are mechanically compressed and melted. The molten polymer is forced through a spinneret as fine jets, with flow speed controlled by the metering pump. The filaments are then cooled and hardened by the quench system to produce fibers [18].

Furthermore, the melt-spinning process is a convenient and straightforward method that does not require coagulation baths or solvents and can produce fibers of varying diameters. Compared to other fiber production methods, it has a simple setup and can be efficiently scaled to create fibers suitable for weaving and usability [19,20,21]. However, this method is primarily used for manufacturing fibers from non-biodegradable synthetic polymers like polyester, polypropylene, and nylon, which can lead to environmental pollution [19,26]. The main reason is that most biodegradable polymers are susceptible to degradation under thermal conditions in this process. On the other hand, fibers cannot be manufactured at a nanoscale via this method, and it is not suitable for applications that need smaller fiber diameters [20].

### 2.4. Wet Spinning

Wet spinning is widely used in textile manufacturing, filtration, healthcare, and industrial sectors [30,31,32]. It involves dissolving a polymer in a solvent to create a spinning solution. This solution is then extruded through spinnerets into a coagulating bath, where the solvent is removed, solidifying the polymer into a fiber. Wet spinning is commonly used for manufacturing fibers from cellulose, acrylics, rayon, and certain synthetic polymers [27]. The main parameters affecting fiber efficiency and quality include the polymer content, extrusion speed, coagulation bath composition and temperature, and drawdown ratio (Figure 2d) [28]. These parameters influence the final mechanical properties and microstructure of the fiber. Although wet spinning allows for the addition of additives to enhance fiber properties and process high-molecular-weight polymers, its slower production speed, complex solvent recovery, and extensive post-treatments limit its environmental sustainability and economic viability [27].

### 2.5. Melt Blowing

Melt blowing is used to produce fibers and nonwoven fabric. In this process, polymer resins are melted at high temperatures and are then extruded through tiny nozzles. A high-speed airstream draws the molten polymer into microfibers (Figure 2e) [29]. These microfibers collect on a moving conveyor belt to form a nonwoven web. The ability to create microfibers makes melt-blowing technology essential across various industries, including automotive, railway, aerospace, agriculture, geotextile, health care, and construction industries, for applications like filtration, medical uses, oil absorption, and thermal insulation [30,31]. The main advantage of this method is its higher production speed and quantity compared to other techniques, along with its low cost and versatility in producing nonwoven fabrics [32]. However, melt-blowing technology has some drawbacks. Most importantly, it can only use thermoplastic polymers. Additionally, controlling fiber size and porosity during production is challenging [33].

### 2.6. Spunbonding

Spunbonding is an efficient method of producing nonwoven fabrics through a continuous process where polymer granules are melted and passed through spinnerets to form fine filaments. These filaments are placed on a conveyor belt and then thermally, chemically, or mechanically bonded to create a strong and flexible fabric (Figure 2f) [29].A key advantage of this technology is its direct conversion from polymer to nonwoven fabric, which eliminates the need for intermediate steps like yarn forming. The main parameters affecting spun fabric quality and properties include polymer type, processing temperatures, spin dimensions, and cooling conditions. Spunbond fabric offers high production speed, cost-effectiveness, and the ability to modify properties such as weight, porosity, and tensile strength to meet specific requirements [22]. However, spunbond fabrics tend to be less soft and flexible than other nonwoven fabrics, making them less suitable for applications that require high drapeability or softness [33]. Other disadvantages include larger average fiber diameters, lack of fiber uniformity, and reduced strength and elongation [22,23,24].

## 3. Mechanical Property Testing

After the selection of the manufacturing method for the desired fibers or mats on the basis of their application, it is crucial to choose the appropriate apparatus for evaluating their durability and strength. This section examines key testing methods, including tensile testing machines, atomic force microscopy (AFM), and dynamic mechanical analysis (DMA), each offering unique insights into the characteristics of biodegradable polymers.

### 3.1. Tensile Testing Machines

The tensile testing machine is a cornerstone in evaluating the mechanical properties of fibrous mats. This device measures the force needed to stretch a fiber until it breaks, providing key parameters such as tensile strength, elongation at break, and Young’s modulus. The fundamental formula used in tensile tests is the stress–strain relationship: stress (σ) equals applied force (F) divided by the original cross-sectional area (A) of the fiber (Equation (1)), whereas strain (ε) is the ratio of extension (ΔL) to the original length (L_0_) (Equation (2)).

(1) $$\sigma =\frac{F}{A},$$

(2) $$\varepsilon =\frac{\Delta L}{L_{0}}$$

Parameters such as force range, extension rate, and stretching speed can be adjusted with this device. These testing machines feature clamps suitable for small fibers and apply tension without shifting or damaging the sample. Thus, they effectively demonstrate how fibers respond to tensile loading and their mechanical behavior in various applications [34,35].

Tensile testing of electrospun fibers and mats typically follows standardized protocols such as ASTM D638, with specimens commonly prepared in rectangular strip or dog-bone shapes to ensure uniform stress distribution and prevent premature failure. For fibrous mats, rectangular strips are often preferred due to ease of handling and consistent load application. In contrast, single fibers require more delicate mounting methods such as adhesive-supported ends or the use of atomic force microscopy.

### 3.2. Atomic Force Microscopy

AFM is a precise technology for probing the mechanical properties of fibers at the nanoscale. It uses a cantilever with a sharp tip that gently contacts the surface of a fiber sample to measure the forces between the tip and the sample, providing detailed nanoscale topography. In addition to mapping, AFM assesses mechanical properties such as elastic modulus, hardness, and adhesion of the fibers. Cantilever deflection is measured as a function of its position across the fiber, following Hooke’s Law:

(3) F=−kx

where F is the force exerted by the tip, k is the cantilever’s spring constant, and x is the cantilever’s displacement. A unique feature of AFM is its ability to image fibers under ambient conditions and in controlled fluid environments, mimicking physiological conditions when necessary. This level of detail makes it an indispensable tool for designing and evaluating advanced fiber materials with tailored mechanical properties [36,37,38].

### 3.3. Dynamic Mechanical Analysis

DMA is an analytical technique used to characterize the mechanical properties of fibers under oscillating forces. It provides critical information about the material’s viscoelastic behavior by capturing its elastic and viscous characteristics. In DMA, stress is applied to a fiber sample while strain, storage modulus, and loss modulus are measured. These parameters indicate the material’s ability to store and dissipate energy, which is vital for applications ranging from industrial textiles to biomedical implants. DMA covers a wide range of frequencies, temperatures, and controlled environments that simulate practical usage conditions. The device’s sensitivity allows it to detect transitions such as the glass transition temperature (Tg) and melting temperature, which are essential for understanding the thermal behavior of fibers in various environments [39,40].

### 3.4. Other Mechanical Tests

In addition to tensile testing, AFM, and DMA, fibers undergo other mechanical tests to evaluate their overall performance. One such test is nanoindentation, which measures hardness and modulus at the nanometer scale. Nanoindentation assesses a nanofiber’s resistance to deformation by pressing a hard indentation into its surface under controlled conditions and measuring the resulting depth. This technique is particularly effective for single nanofibers, where other methods may be inadequate [10]. Another important test is microcompression, which compresses a nanofiber to determine its buckling behavior. This test is crucial for applications involving high-pressure loading [10,41].

## 4. Mechanical Properties of Biodegradable Polymer Fibers and Mats

This section presents a comparative overview of the mechanical properties of various biodegradable fibers and mats, highlighting how different material types and processing methods influence their strength, flexibility, and durability. Key factors discussed include polymer type, fabrication technique, and pretreatment or post-treatment strategies used to enhance alignment, crystallinity, or interfacial bonding. The discussion is organized into sections on natural and synthetic biodegradable polymers to highlight their respective advantages, challenges, and performance trends in fiber-based systems. This section aims to guide researchers in selecting appropriate polymer types and processing techniques to improve the mechanical performance of biodegradable fibers and fibrous mats for specific applications.

### 4.1. Synthetic-Biodegradable-Polymer-Based Fibers

#### 4.1.1. Polyvinyl Alcohol

Polyvinyl alcohol (PVA) is a synthetic, biodegradable, biocompatible, nontoxic, hydrophilic, and water-soluble polymer obtained by the hydrolysis of polyvinyl acetate (C_4_H_6_O_2_)_n_. The crystallinity and polarity of PVA are two key factors that can be affected by the degree of hydrolysis of PVA. A higher degree of hydrolysis increases crystallinity because of a greater number of polar hydroxyl groups (–OH) [42,43]. As crystallinity increases, molecules become more tightly packed together in a higher order. This improved distribution and ability to resist applied forces result in higher stiffness and tensile strength [44].

PVA enhances the mechanical properties, biodegradability, biocompatibility, hydrophilicity, and processability of other polymers [42]. Its excellent physical properties, low cost, biodegradability, and biocompatibility make it widely used in medical applications, packaging, adhesives, wound dressings, drug delivery systems, soft contact lenses, and artificial organs [43,45,46]. PVA’s biodegradability stems from its susceptibility to degradation by microbial action, where microorganisms like bacteria and fungi can break down the polymer’s molecular chains using enzymes such as PVAase, hydrolyzing it into simpler, water-soluble compounds. Thus, this microbial susceptibility, combined with its water solubility and hydrolyzable backbone, enables PVA to degrade effectively in a range of natural environments [47,48,49].

**Figure 3:**
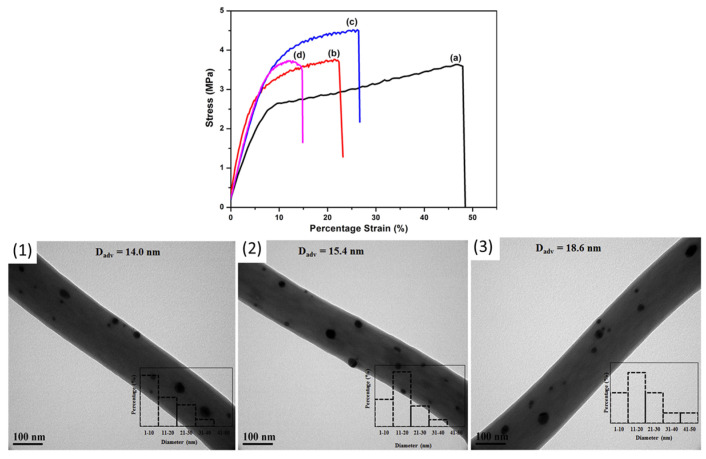
Stress–strain curves of hybrid nanofibers (PVA/chitosan) with different AgNP contents: (a–d) 0, 0.25, 0.5, and 1 wt.%, respectively. Images (1), (2), and (3) show the TEM morphology of nanofibers containing AgNPs at 0.25, 0.5, and 1 wt.%, respectively [51]. (reprinted with permission).

Electrospun PVA-based fibers exhibit tunable mechanical properties that vary depending on nanoparticle incorporation, polymer blending, and processing conditions. For instance, adding cellulose nanowhiskers can improve tensile properties, especially in aligned fibers where the nanowhiskers orient along the fiber axis, enhancing stress transfer and reinforcing the polymer matrix. Tensile strength in aligned fibers increased from 5.37 to 10.5 MPa, demonstrating effective nanowhisker reinforcement [50]. Incorporating silver nanoparticles (AgNPs) can initially strengthen PVA/chitosan fibers because of the strong intermolecular interactions (Figure 3). However, at higher concentrations, AgNPs aggregate, disrupting uniform distribution and reducing mechanical performance. This aggregation hinders polymer chain mobility, increasing stiffness but decreasing flexibility [45]. The highest tensile strength of 4.52 MPa was achieved at 0.5% AgNP content before declining at higher concentrations [51].

Polymer blending also significantly influences mechanical behavior. For example, blending PVA with soy protein isolate (SPI) has been shown to weaken fiber strength, as the globular structure of SPI disrupts the continuity of the PVA matrix and hinders efficient chain entanglement. This incompatibility at the molecular level reduces stress transfer across the fiber network, leading to decreased tensile strength and elongation [52]. In contrast, incorporating silk sericin (SS) and applying thermal treatment strengthens the fibers by promoting hydrogen bonding, which improves molecular cohesion and elasticity. Heated SS/PVA fibers exhibit over five times higher tensile strength than untreated fibers, increasing from 0.7 to 3.7 MPa, highlighting the role of thermal treatment in enhancing mechanical properties [46].

Structural modifications such as porosity control and plasticizer addition further affect fiber properties. Increased porosity in PVA/γ-Fe_2_O_3_ nanofibers weakens fiber interactions, reducing tensile strength. Similarly, although plasticizers enhance flexibility by disrupting internal hydrogen bonding, they also reduce stiffness by altering molecular packing. In melt-spinning studies of PVA, the addition of plasticizers has been shown to increase tensile strength by nearly three-fold, while reducing elongation due to alterations in hydrogen bond distribution [53].

The influence of water content in melt-spun PVA fibers has shown how processing parameters control mechanical results. Lower water content restricts molecular movement because of stronger hydrogen bonding, making fibers stiffer but less stretchable. In contrast, higher water content increases chain mobility, enhancing flexibility while reducing overall strength. In one study, the yield stress of PVA fibers rose from 17 to 109 MPa as water content decreased, emphasizing hydrogen bonding’s role in regulating stiffness [54]. Therefore, achieving optimal mechanical performance in PVA fibers requires balancing nanoparticle dispersion, polymer compatibility, structural modifications, and processing conditions to tailor strength, stiffness, and flexibility for specific applications.

#### 4.1.2. Polyethylene Oxide

Poly (ethylene oxide) (PEO) is a water-soluble biodegradable synthetic polymer consisting of a long chain of ethylene oxide units that are chemically linked. Its ability to break down into nontoxic by-products (ethylene glycol and water) makes it suitable for various pharmaceutical and biomedical applications [55]. PEO is a commonly used sacrificial porogen that can be easily electrospun and rapidly dissolved in aqueous solutions [56,57].

Merchiers et al. [17] compared the mechanical properties of PEO fibers produced by centrifugal force spinning (CFS) and electrospinning. CFS fibers exhibited higher tensile strength (1.8–4.2 MPa) and greater elongation at break than electrospun fibers (0.2–2.4 MPa), likely because of increased crystallinity. Blending PEO with carboxymethyl chitosan (CMCS) in centrifugal spinning reduced elongation (from 22.8% to 11.2%) but increased Young’s modulus (from 10.5 to 26.6 MPa), indicating a stiffening effect. However, tensile strength fluctuated (0.8–2.2 MPa), suggesting complex interactions between CMCS and PEO [58]. Incorporating PEO into starch-based fibrous membranes enhanced tensile strength approximately three-fold (from 0.39 to 1.8 MPa) because of improved polymer chain entanglement [59].

Kupka et al. [57] examined electrospun PEO/PCL nanofibers and found that blending hydrophilic PEO with hydrophobic PCL significantly increased Young’s modulus. Mats with 75/25 and 50/50 PCL/PEO compositions showed a 2.5-fold increase in stiffness relative to pure PCL and PEO. The improved mechanical properties may result from interpenetrating networks formed at the nanoscale. Overall, PEO fibers produced via CFS exhibit better tensile strength and elongation than electrospun fibers. Blending with CMCS increases stiffness while reducing flexibility, whereas incorporating PEO into starch-based matrixes strengthens fiber networks. PEO/PCL blends significantly enhance mechanical properties because of nanoscale interpenetrating networks, making them promising for structural applications.

#### 4.1.3. Polyglycolic Acid (PGA) and Poly(lactic-co-glycolic acid) (PLGA)

Polyglycolic acid (PGA) is a synthetic, semicrystalline, biodegradable polyester that degrades into nontoxic by-products, such as carbon dioxide and water, over several weeks under physiological conditions. Its high tensile strength, stiffness, and biocompatibility make it primarily suitable for biomedical sutures, reducing the need for removal surgeries [60,61]. Its copolymer, poly(lactic-co-glycolic acid) (PLGA), derived from lactic acid and glycolic acid, features tunable degradation rates and mechanical properties, making it widely used in drug delivery systems, tissue engineering, and biomedical devices. PLGA’s ability to degrade into nontoxic components enhances its appeal for medical applications [60,62].

Researchers have explored blending PGA with PLA to address PGA’s limitations, including high crystallinity, rapid degradation, and poor processability. Ramdhanie et al. [63] demonstrated that adjusting the PGA-to-PLA ratio in electrospun nanofibers allowed for tuning mechanical properties to match those of collagen and elastin, key structural components in soft tissues. The modulus ranged from 3.4 to 140 MPa, tensile strength from 2.2 to 11 MPa, and strain at break from 70% to 330%, highlighting the potential of PGA/PLA blends for soft tissue engineering.

Comparative studies of synthetic biodegradable polyesters found that PLGA (50/50) exhibited the highest Young’s modulus (144 MPa), followed by PGA (138 MPa) and PLGA (85/15) (114 MPa). PCL and PLLA showed the lowest moduli, around 8.5 MPa, indicating that PGA retains relatively high stiffness among biodegradable polymers [64].

**Figure 4:**
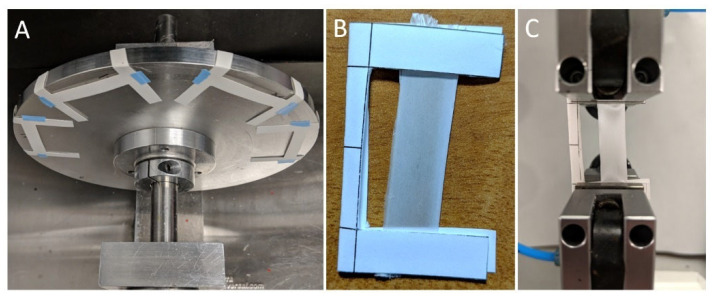
Electrospinning and tensile testing setup: (**A**) rotating mandrel for fiber collection with I-frames; (**B**) I-frame with fiber mat secured for tensile testing; and (**C**) a fiber mat in Test Resources universal testing system grips before testing [65] (reprinted with permission).

D’Amato et al. [65], as shown in Figure 4, examined the effect of residual electrospinning solvent on PGA fibers and found that solvent retention initially softened the fibers but led to increased brittleness over time. Young’s modulus nearly tripled from 1013.4 to 2989.1 MPa within 14 days as the solvent evaporated, while yield strain decreased from 3.5% to 1.5%, demonstrating that solvent removal significantly affects the mechanical performance of PGA scaffolds.

To enhance porosity, researchers investigated electrospraying sacrificial PEO microparticles into PGA, PLGA, and PCL scaffolds. This technique improved porosity and cell infiltration while maintaining the mechanical integrity of PGA and PLGA. However, the removal of PEO microparticles reduced the strength and stiffness of the more elastic PCL component, indicating its greater sensitivity to porosity modifications [56].

The mechanical properties of PLGA/PCL blends were investigated through coaxial electrospinning. Bazgir et al. [66] reported that coaxial scaffolds with PCL as the core and PLGA as the sheath had lower tensile strength (3.1 vs. 2.89 MPa) and elongation (25.94% vs. 14.61%) compared to pure PLGA membranes. PCL scaffolds exhibited the lowest tensile strength (1.02 MPa) but the highest elongation at break (32.43%). Further studies showed that increasing PLGA content enhanced the tensile strength of PLGA–PCL blends [67,68]. Overall, PGA-based fibers demonstrate high stiffness, with solvent evaporation significantly affecting their mechanical behavior. Blending PGA with PLA can tailor its mechanical and degradation properties, making it more suitable for soft tissue engineering, while PLGA/PCL blends allow for tunable flexibility and strength, which are advantageous in load-bearing or long-term implants.

#### 4.1.4. PLA, Poly(L-lactic acid) (PLLA), and Poly(D-lactic acid) (PDLA)

PLA and PLLA are aliphatic polyesters derived from lactic acid (2-hydroxy propionic acid) monomers. PLA is a copolymer containing L- and D-lactic acid units, whereas PLLA is a homopolymer consisting solely of L-lactic acid units. This difference affects their chain’s structural conformation, properties, and specifications [69]. They can be easily spun and are suitable for packaging, agriculture, and particularly biomedical applications, including sutures, orthopedic implants, and drug delivery systems [70]. The biodegradability of PLA and PLLA stems from their ability to be metabolized by the human body. Their hydrolytic degradation kinetics produce lactic acid, which can be converted to carbon dioxide and water. Their polyester backbone can undergo hydrolytic cleavage under physiological conditions, breaking down into lactic acid, a natural product of human metabolism. This property is highly desirable in the biomedical field, as it eliminates the need for additional surgeries to remove temporary implants [71,72]. Because of their thermoplastic processability, biological properties, biodegradability, and lack of immunogenicity, they are suitable for tissue engineering. However, PLA-based fibers often exhibit low hydrophilicity and inherent brittleness, which can hinder their interaction with cells in soft tissue applications. Therefore, there is considerable interest in increasing hydrophilicity and reducing the brittleness of PLA-fiber-based scaffolds. For example, adding soft and hydrophilic PEG polymer to the PLA solution can enhance surface hydrophilicity [72].

The mechanical properties of electrospun PLLA fibers depend highly on solvent composition and surfactants. Arbeiter et al. [73] demonstrated that adding surfactants, such as TEAC, formic acid, and Triton X-100, influenced tensile strength and crystallinity. TEAC produced the most significant increase, raising tensile strength from 2.7 to 27.1 MPa, whereas TX-100 improved flexibility but slightly reduced elongation, indicating that surfactants alter molecular interactions and fiber structure.

Crosslinking can also enhance mechanical properties. Qiao et al. [74] found that adding dicumyl peroxide (DCP) and triallylisocyanurate (TAIC) to PLLA fibers increased rigidity while maintaining some flexibility, increasing the modulus from 165 to 208 MPa. This improvement demonstrates the potential of chemical modifications in tailoring electrospun PLLA properties for biomedical applications. Additionally, Goreninski et al. [75] investigated iodine and iodine-containing compounds, traditionally used as antiseptics, in PLLA and PCL fibers. Their findings showed that iodine treatment did not significantly affect the scaffolds’ mechanical properties or morphologies.

Furthermore, without altering the underlying chemistry, post-treatment of electrospun PLLA mats can significantly enhance mechanical performance. Immersion in acetone promotes the growth of α-form crystals, approximately doubling the tensile strength and increasing the Young’s modulus, while reducing extensibility [14]. A similar acetone-based rolling treatment applied to tubular PLLA grafts increases the wall strength from sub-megapascal values to over 5 MPa, in line with crystallinity gains confirmed by XRD and DSC [76]. Post-treatment strategies that control porosity yield comparable outcomes. Porous PLLA membranes, prepared by soaking either neat PLLA fibers or PLLA/PCL blends, show steeper stress–strain curves and lower elongation at break. These effects result from a combination of increased crystallinity and fiber–fiber welding [1,77]. Overall, solvent-based treatments that increase the amount and quality of α-phase crystals can effectively improve strength and stiffness, but they often reduce flexibility. This trade-off should be considered when designing PLA-based materials.

Material additives such as bioceramics, essential oils, and nanoparticles also influence fiber properties. Didekhani et al. [78] incorporated oyster shell (OS) particles into PLLA nanofibers, which increased tensile strength while reducing elongation at break. Post-plasma treatment enhanced surface hydrophilicity, making the scaffold more suitable for tissue engineering [78]. Similarly, PLA fibers blended with rosmarinic acid (RosA) and graphite oxide (GO) increased breaking strength from 0.8 to 2.6 MPa but became more brittle because of the reduced fiber diameter and increased crystallinity [79].

Inorganic bioceramic fillers such as hydroxyapatite (HA), amorphous calcium phosphate (ACP), and β-tricalcium phosphate (β-TCP) influence both mechanical properties and bioactivity of PLA/PLLA fibers. For example, adding 5 wt% HA to electrospun PLLA reduces tensile strength from 4.9 to 3.2 MPa due to stress concentration but enhances apatite formation [1]. Similarly, porous PLLA/ACP mats show a three-fold increase in strength after acetone treatment, along with improved modulus and cell response. PLA fibers coated with β-TCP show enhanced osteogenic activity under cyclic loading, although mechanical values are not reported [80]. These examples demonstrate the dual mechanical and biological effects of calcium phosphate-based fillers in PLA systems.

The incorporation of nanocrystalline cellulose (NCC) improved PLA fiber strength, nearly doubling tensile properties at 2% NCC loading, whereas lignin addition enhanced cell proliferation without significantly altering mechanical behavior [81].

**Figure 5:**
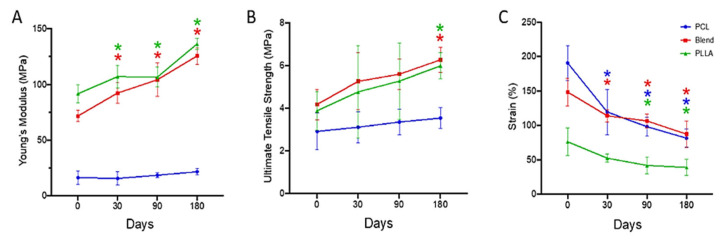
Mechanical properties of PCL, 50:50 PCL: PLLA blend, and PLLA electrospun fibers following hydrolytic degradation in phosphate-buffered saline solution at 37 °C; (**A**) Young’s modulus, (**B**) ultimate tensile strength, and (**C**) percentage strain [82]. Stars (*) represent statistical significance compared to non-degraded scaffold (time zero). (reprinted with permission).

Environmental factors, such as hydrolytic degradation, also impact the mechanical properties of PLA and PLLA fibers. Shamsah et al. [82] found that hydrolysis over six months increased crystallinity, making fibers stiffer and stronger but also more brittle (Figure 5). Ultimate tensile strength increased by 75% for PLA-PLLA blends because of polymer chain rearrangement, demonstrating how controlled degradation can enhance mechanical performance in biodegradable materials.

The fabrication method significantly influences the mechanical properties of PLA nonwoven materials. Feng et al. [83] investigated a melt-blowing system for producing PLA nonwoven mats, highlighting how the die-to-collector distance (DCD) affects material properties. Reducing the DCD from 200 to 75 mm decreased the fiber diameter, resulting in a denser structure with improved fiber bonding. Consequently, the tensile strength more than doubled to 0.18 MPa, attributed to increased web density and stronger fiber interactions.

Similarly, Zhou et al. [84] examined the effect of centrifugal melt-spinning speeds on PLA fiber properties. They found that higher spinning speeds refined the fiber structure, enhancing tensile strength and modulus. At 1500 rpm, tensile strength reached 3.31 MPa, and the elastic modulus significantly improved to 66.77 MPa. These results are consistent with previous studies, including that of Boschetto et al. [85], which reported a tensile strength of 3.3 MPa at a higher speed of 2200 rpm, demonstrating a strong correlation between processing speed and mechanical performance.

Additionally, the durability of PLA fibers is often evaluated using tenacity, which measures the breaking force relative to linear density. Chen et al. [86] produced PLA fibers using melt spinning at 800 m/min, reporting a tenacity of 3.7–4.7 cN/tex, making PLA a competitive alternative to other synthetic polymers. Hassan et al. [87] further showed that PLA fibers have an initial modulus of 80.43 cN/tex and an elongation at break of 19%, reinforcing their suitability for various textile applications [19,83].

Beyond PLLA, recent studies have highlighted the distinct mechanical behavior of its enantiomer, PDLA, under similar processing conditions. Published data confirm that PDLA exhibits higher strength and modulus than its L-enantiomer under equivalent spinning conditions. As-spun PDLA nanofiber mats display a tensile modulus of approximately 47 MPa and a maximum tensile strength of around 3 MPa. Brief annealing increases the strength to 4–5 MPa, although stiffness decreases due to simultaneous homocrystallization and chain relaxation [88]. Conjugate electrospinning of continuous yarns further elevates the tensile strength to about 285 MPa, reflecting the higher crystallinity achievable with the D-isomer [89]. When PDLA is involved in stereocomplex formation either as a coaxial shell or as a 1:1 blend with PLLA, the mechanical behavior diverges. Interfacial stereocomplexes tend to soften the fibers (modulus ≈ 20 MPa, elongation at break ≈ 30 percent), whereas hybrid yarns or membranes can exhibit up to a 30 percent increase in tensile strength compared to the homopolymer [88,90]. These observations highlight that mechanical performance is influenced more by the spatial distribution of stereocomplex crystallites than by the overall D-content alone. Optimizing the mechanical properties of PLA and PLLA fibers involves balancing solvent choice, crosslinking, nanoparticle incorporation, degradation behavior, and processing parameters to achieve targeted strength, flexibility, and durability.

#### 4.1.5. PCL

Polycaprolactone (PCL) is a synthetic aliphatic biodegradable polyester approved by the FDA for human use, with a low melting point of around 60 °C and a glass transition temperature of about −60 °C. This semicrystalline polymer can enhance the biodegradability of other polymers and can be processed via methods such as electrospinning, injection molding, extrusion, and 3D printing because of its excellent melt processing and processability. PCL has a high degree of elongation, adequate tensile strength, and very low solubility in water [91], which are essential for controlled release in biomedical applications [92]. These properties make PCL nanofibers suitable for long-term implantable systems and drug delivery [93,94].

PCL’s biodegradability stems from its aliphatic polyester structure, which degrades into nontoxic products under physiological conditions through hydrolysis of its ester bonds. It is well known for its good biocompatibility. Its relatively low degradation rate of up to 3–4 years, compared to other biopolymers like PLA, allows for extended application, making it particularly useful in the medical sector, where prolonged support and gradual biodegradation are desired [93,95].

PCL is widely regarded and frequently combined with PLA or PLLA given its excellent mechanical and viscoelastic properties, high cytocompatibility, and slow degradation rate. These characteristics have made PCL a staple in biomedical engineering, and it has been approved by the FDA for clinical applications [96,97].

Solvent systems, processing techniques, and material modifications highly influence the mechanical properties of PCL fibers. Sharma and Satapathy [98] investigated how solvent conductivity affects electrospun PLA-PCL fibers, finding that PLA fibers spun in a chloroform/DMF system exhibited higher stiffness, whereas PCL fibers showed greater flexibility. A 70/30 PLA-PCL blend in a dichloromethane/DMF system demonstrated an improved strain at break of 155%, highlighting how solvent–polymer interactions impact fiber mechanics. These findings align with trends observed in other studies that have examined the addition of PCL to PLA or PLA composites, further emphasizing the advantages of including PCL in these blends [96,97,99,100].

Also, beyond solvent choice, several additional variables influence the mechanical behavior of PCL-containing fibers. First, the blend ratio plays a key role: adding PCL to PLLA lowers modulus and tensile strength but increases ductility, with elongation rising from 59 percent in neat PLLA to 87–132 percent as the PCL content increases from 10 to 30 weight percent. Second, post-treatment with acetone for five minutes induces recrystallization of PLLA, partially fuses adjacent fibers, and triples the Young’s modulus while reducing elongation. Third, the same treatment creates surface porosity and drives PCL chains toward the fiber surface, lowering the water contact angle from 134° to 112°, enhancing cell affinity, and increasing stiffness through shell-like crosslinks. Together, blend composition, solvent-driven crystallinity, and morphological evolution interact with solvent–polymer compatibility to tune the performance of PCL-rich fiber mats [1]. Other studies in which PCL is blended with other polymers are discussed in other polymer sections.

**Figure 6:**
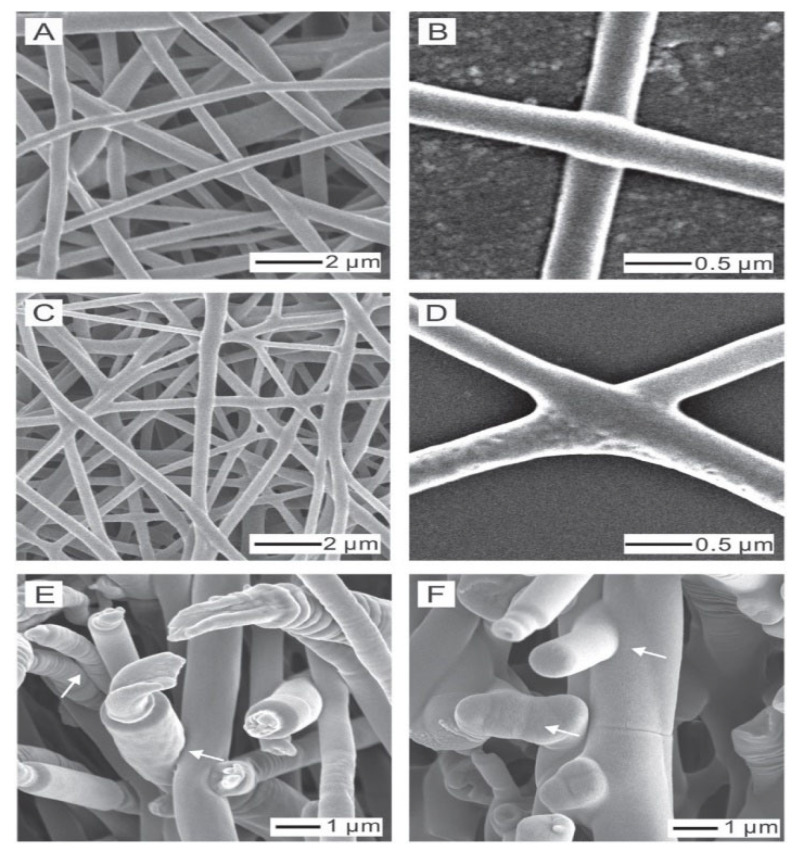
SEM images of electrospun PCL nanofiber mats exposed to 25 μL of DCM vapor in a closed vial for durations of (**A**,**B**) 30 min and (**C**,**D**) 60 min; (**E**) cross section of nanofibers before treatment (arrows show no interfiber bonding); (**F**) cross section of nanofibers after 60 min (arrows indicate welding at cross points) [102] (reprinted with permission).

Surface modifications significantly alter mechanical behavior. Volokhova et al. [101] examined the effects of pulsed electron beam (E-beam) irradiation on PCL nanofibers. Although elongation remained unchanged, the treatment reduced tensile strength from 4.1 to 1.3 MPa and modulus from 48 to 5 MPa because of polymer chain destruction. In contrast, using another surface modification method, Li et al. [102] enhanced PCL fiber mechanics through solvent vapor welding as shown in Figure 6, which restricted nanofiber slippage and increased tensile strength from 11.53 MPa to 21.4 MPa, demonstrating how structural reinforcement improves mechanical performance.

Nanoparticle incorporation further refines fiber properties. Rajzar et al. [103] found that adding hydroxyapatite (HAp) to electrospun PCL scaffolds enhanced bioactivity but slightly reduced tensile strength because of particle-induced brittleness. Similarly, Khunová et al. [104] showed that halloysite nanotubes (HNTs) doubled tensile strength and significantly improved elongation and thus enhanced the interfacial interactions of fiber networks.

Using the AFM method to evaluate the mechanical properties of individual fibers, Baker et al. [36] observed that electrospun PCL nanofibers could stretch up to 98% before slipping, achieving a tensile strength of at least 12 MPa. Similarly, Adrian et al. [105] reported that combining PCL fibers with HAp increased Young’s modulus from 4 to 20 MPa, highlighting the reinforcement effect of ceramic fillers.

Processing parameters, molecular weight, and fabrication methods influence PCL fiber performance. Alharbi et al. [93] found that fiber modulus varied between 257 and 340 MPa, depending on molecular weight and fiber diameters. Loordhuswamy et al. [106] showed that blending PCL with gelatin increased modulus from 40.76 to 84.87 MPa, although tensile strength declined because of phase separation between hydrophobic and hydrophilic polymers. Overall, selecting appropriate solvent systems, reinforcement strategies, and processing techniques can tune the mechanical properties of PCL fibers to balance flexibility, strength, and bioactivity for specific applications.

Table 1 presents a detailed overview of mechanical enhancement strategies applied to synthetic biodegradable polymers. It highlights various approaches, including nanoparticle addition, copolymerization, crosslinking, and processing modifications. For each method, the table specifies the polymer system, improvements in fiber diameter and mechanical performance (such as tensile strength and elongation), and related applications, along with supporting references.

### 4.2. Natural-Biodegradable-Polymer-Based Fibers

#### 4.2.1. Chitosan

Chitosan (CS), or poly [β-(1-4)-2-amino-2-deoxy-D-glucopyranose], is the second most abundant natural polysaccharide after cellulose, obtained from the deacetylation of chitin, primarily derived from crustacean shells [51,108,109]. Its biodegradability, biocompatibility, nontoxicity, and antibacterial, antifungal, and antioxidant properties make it an excellent biopolymer for pharmaceuticals, nutrition, agriculture, food treatment, cosmetics, smart materials, and biomedicine [110,111].

Chitosan can be easily degraded by chitinase and chitosanase, which are found in nature, breaking it down into simpler, water-soluble compounds that microorganisms can ingest. Consequently, chitosan degrades into nontoxic materials, such as carbon dioxide and water. However, the relatively weak mechanical properties of chitosan fibers remain a primary concern [109]. Chitosan is often combined with other natural or synthetic polymers to address this concern and enhance its mechanical properties and processability [42].

**Figure 7:**
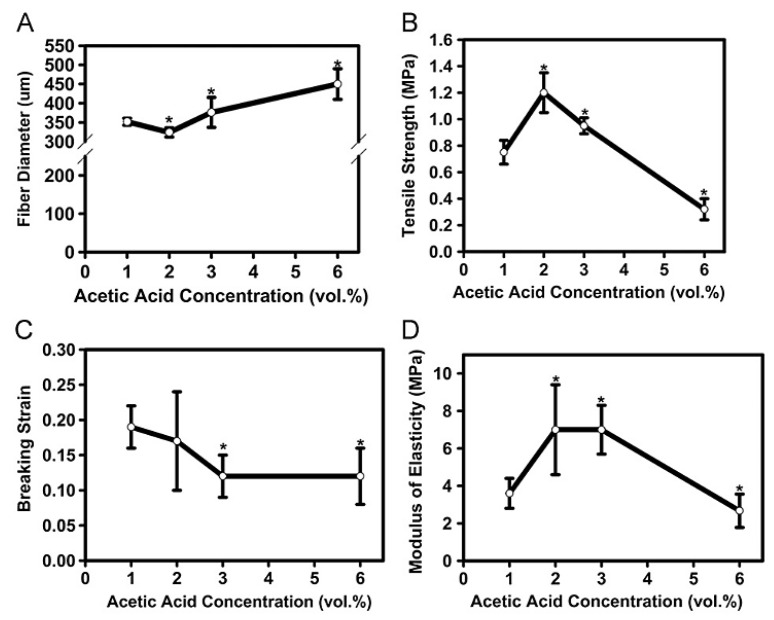
Effect of acetic acid concentration on the (**A**) diameter, (**B**) strength, (**C**) elasticity, and (**D**) stiffness of chitosan fibers [109] (reprinted with permission). “*” indicates *p* < 0.05 compared the 1 vol% AA.

Solvent concentration, thermal treatments, crosslinking, and nanoparticle incorporation influence the mechanical properties of chitosan fibers. Albana et al. [109] examined how acetic acid and ammonia modified fiber strength and modulus (see Figure 7 for the effects of acetic acid). Higher acetic acid concentrations initially enhanced tensile strength because of improved solubility and crystallinity, but concentrations beyond 2 vol.% caused excessive swelling that weakened fiber structure. Ammonia neutralization reinforced fiber rigidity, but excessive concentrations reduced elasticity, demonstrating how solvent interactions alter chitosan’s mechanical behavior.

Barnes et al. [112] incorporated polydioxanone (PDO) with chitosan and found that collagen type I increased stiffness compared to type III. The resulting fibers closely mimicked native extracellular matrix structures, making them promising for vascular graft applications.

Crosslinking with pullulan and cinnamaldehyde further improved chitosan fiber properties. Increasing the chitosan-to-pullulan ratio raised tensile strength from 11.36 to 22 MPa and significantly enhanced modulus, indicating improved polymer network interactions. However, reduced elongation at break suggested that rigid crosslinking limited fiber flexibility [113].

The incorporation of nanoparticles into the fibers has also enhanced their mechanical properties. Lue et al. [51] demonstrated that embedding silver (Ag) nanoparticles in a chitosan–PVA matrix increased tensile strength from 3.65 to 4.52 MPa at optimal loading, attributed to strong intermolecular interactions [42]. In contrast, ceramic nanoparticles like ZnO and TiO_2_ showed lower dispersion efficiency, emphasizing the importance of nanoparticle–polymer compatibility in optimizing fiber strength [114,115]. Overall, solvent processing, polymer blending, and nanoparticle incorporation provide tunable pathways for enhancing chitosan fiber mechanical properties, balancing strength, flexibility, and bioactivity for biomedical applications.

#### 4.2.2. Collagen

Collagen is a natural polymer present in almost every tissue, maintaining biological and structural integrity [116]. Its biodegradability stems from collagen’s unique amino acid sequence, recognized and processed by various enzymes and microorganisms in the body. Enzymes like collagenases and proteases break down collagen into smaller peptides and amino acids, which can be reabsorbed for metabolic processes or expelled harmlessly [117]. Because of its advantageous properties that match extracellular matrix (ECM) structural proteins, collagen is widely used in biomedical applications, such as tissue-engineered scaffolds, wound dressings, and drug delivery systems [117,118].

The mechanical properties of collagen fibers can be improved through polymer blending, crosslinking, and enzymatic modifications. Blending collagen with PCL enhances fiber strength and stability, resulting in a collagen–PCL fibrous mesh with a tensile strength of 2.5 MPa. Thicker fibers contribute to higher strength because of a better alignment [116]. Chandika et al. [118] developed bilayer scaffolds with fish collagen (FC) and PCL, demonstrating that increasing PCL content significantly improved tensile strength to 12.61 MPa at 90% PCL concentration, attributed to enhanced polymer interactions and structural reinforcement.

Mechanical stability can also be tailored through enzymatic degradation. Panwar et al. [119] investigated how cysteine protease (catK) affected collagen fiber integrity and observed that Young’s modulus decreased from 3.2 to 1.9 GPa because of disrupted fibrillar bridges. This enzymatic weakening illustrates how degradation processes influence collagen’s structural cohesion.

Overall, collagen-based fibers exhibit tunable mechanical properties through polymer reinforcement and biochemical modifications, facilitating applications in tissue engineering and biomaterials.

#### 4.2.3. Cellulose

Cellulose, the most abundant biopolymer, is known for its exceptional biodegradability, hydrophilicity, and extensive chemical modification capacity, formed by a linear polysaccharide. Cellulose is susceptible to enzymatic hydrolysis by cellulases produced by various microorganisms, including bacteria and fungi, that break down cellulose into glucose molecules for energy. Cellulose-based electrospun fibers have been widely explored for applications in tissue engineering scaffolds, wound dressings, filtration, energy storage, and composite reinforcement because of their high surface area, porosity, and structural similarity to the extracellular matrix. Furthermore, cellulose can be chemically modified to produce derivatives such as cellulose esters (e.g., acetate, nitrate), cellulose ethers (e.g., methyl cellulose, carboxymethyl cellulose), cellulose nanocrystals/nanofibrils, and cellulose-based composites, with applications in diverse industries [120].

The mechanical properties of cellulose-based fibers depend on fiber architecture, polymer blending, and nanoparticle incorporation. Coaxial electrospinning can improve fiber strength by creating core–shell structures. For instance, cellulose acetate (CA) fibers reinforced with a PCL core exhibited a four-fold increase in tensile strength, attributed to improved load distribution and polymer compatibility [121].

Cellulose nanocrystals (CNCs) have also been explored for reinforcement. Rahmat et al. [18] incorporated CNCs into PLA fibers and then applied a hot water treatment to improve fiber stiffness and tensile strength because of their enhanced crystallinity. Similarly, Shi et al. [122] demonstrated that increasing CNC concentration to 5% significantly improved tensile stress and modulus; however, concentrations greater than 10% led to CNC aggregation and thus reduced fiber integrity. Incorporating CNCs into PCL fibers also improved mechanical properties, with optimal reinforcement at 10% CNC loading. Beyond this concentration, tensile modulus peaked at 7.2 MPa, but excessive CNC content led to nanoparticle aggregation, reducing fiber flexibility [123].

Surface modifications have also been investigated. PLA fibers coated with cellulose nanowhiskers (CNWs) exhibited up to a 45% increase in tensile modulus because of strong CNW–CNW interactions that improve fiber rigidity without significantly altering tensile strength [124].

**Figure 8:**
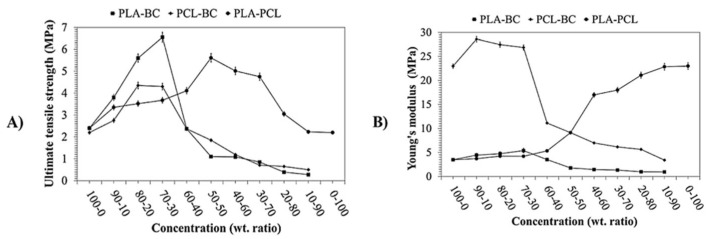
Tensile results of the ternary sample (PLA-PCL)-BC: (**A**) ultimate tensile strength of the binary systems and (**B**) Young’s modulus of the binary systems [125] (reprinted with permission).

Blending bacterial cellulose (BC) with PCL and PLA via centrifugal spinning demonstrated how composite structures can enhance fiber strength, as shown in Figure 8. The optimized ternary PLA-PCL-BC blend exhibited a tensile strength of 9.1 MPa, balancing the elasticity of PCL with the high mechanical strengths of PLA and BC [125]. In summary, cellulose-based fibers achieve improved mechanical properties through core–shell structures, CNC reinforcement, and BC blending, offering versatile applications in bio-based and high-performance materials.

#### 4.2.4. Starch

**Figure 9:**
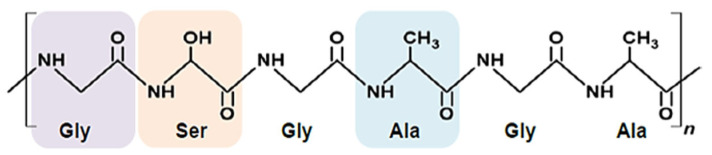
The chemical structures of silk fibroin [131] (reprinted with permission).

Starch, the second most abundant polysaccharide, is found in many plants, including potatoes, as well as tapioca and cornstarch [126,127]. It has diverse applications in food products, packaging, filtration, drug delivery, composites, and textiles, given its cost-effectiveness, biodegradability, and renewability [128,129]. Starch consists of amylose (a linear polymer of α-(1–4) glucose units) and amylopectin (a branched polymer of α-(1–4) glucose units with intermittent divisions of α-(1–6) connections). The presence of intermolecular and intramolecular hydrogen bonds complicates the thermoplastic processing of starch (Figure 9) [129]. Studies on plasticization, as well as physical and genetic modifications, have aimed to enhance the mechanical and functional properties of starch for industrial use. Blending starch with other biodegradable polymers has shown promise in producing materials with improved strength, flexibility, and processability. Its simple structure, composed of glucose units, allows easy breakdown by enzymes from microorganisms, enabling utilization as a carbon and energy source [127].

The mechanical properties of starch-based fibers largely depend on processing techniques and polymer blending. The strong intramolecular hydrogen bonds of starch complicate its processability, but Lancuški et al. [130] demonstrated that formic acid effectively disrupts starch granules, enhancing solubilization and esterification. Electrospun fibers from pure starch in formic acid exhibited a Young’s modulus of 264 MPa, highlighting the role of acid treatment in improving fiber structure.

Blending starch with PVA has been explored to enhance mechanical performance. Afzal et al. [128] showed that increasing PVA content significantly improved tensile strength to 11.54 MPa, owing to PVA’s carbon–carbon backbone and hydroxyl interactions. This approach compensates for starch’s fragility, making it suitable for applications requiring improved mechanical integrity.

#### 4.2.5. Silk Fibroin

Silk fibroin (SF) is a structural protein derived from the silk fibers of domesticated *Bombyx mori* (*B. mori*), with a content of the amino acids alanine, glycine, and serine reaching up to 90%, and can be decomposed by enzymes (Figure 9).

Silk fibroin is typically extracted from degummed *Bombyx mori* silk by dissolving it in either 9.3 M LiBr at 60 °C followed by dialysis or in a hot CaCl_2_/ethanol/water mixture (1:2:8) followed by dialysis and freeze-drying. These methods yield aqueous silk solutions or powders suitable for further processing [132]. It consists of a fibrous protein called fibroin in the core and glue-like proteins called sericin that cover the fibroin fibers [133]. SF has several beneficial properties, including good biocompatibility, satisfactory toughness, elasticity, minimal inflammatory reaction, and biodegradability, making it suitable for tissue engineering, drug release, and regenerative medicine. It has long been recognized as a high-quality textile fiber and suture material [134].

The mechanical properties of SF stem from its crystallinity and molecular conformation. To enhance the mechanical properties of electrospun silk fibers, researchers use post-processing techniques to induce a conformational transition from random coils to β-sheets [135]. This process converts the random coil conformation of SF into a β-sheet conformation through treatments with organic solvents, such as methanol. Consequently, methanol-treated SF exhibits significantly increased crystallinity and greater dimensional stability [133].

The mechanical properties of silk SF fibers are affected by post-treatment methods, fiber alignment, and polymer blending. Methanol treatment effectively enhances fiber strength by increasing β-sheet content and reinforcing interchain interactions. Amiraliyan et al. [135] reported that methanol-treated SF fibers achieved a tensile strength of 28.53 MPa, whereas elongation at break decreased because of the increased stiffness of the structure.

**Figure 10:**
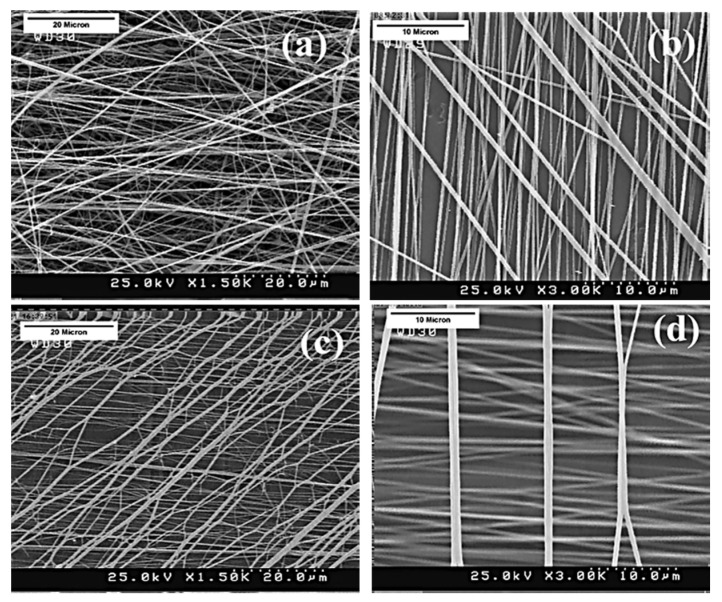
SEM micrograph of electrospun silk fibroin (SF) fibers: (**a**) random orientation; (**b**) SF 60 with knitted orientation (central angle 60°); (**c**) SF 60 with knitted orientation (central angle 60°), fixed by methanol immersion; (**d**) SF 90 with knitted orientation (central angle 90°) [134] (reprinted with permission).

Further investigations have analyzed the impact of methanol treatment on various fiber configurations. Dodel et al. [134] examined three scaffold structures: random orientation and knitted structures with 60° and 90° interstitial angles. Although methanol treatment did not significantly alter fiber surface morphology, it greatly improved mechanical strength from 4.4 to 15.3 MPa (Figure 10). This improvement was attributed to increased β-sheet formation, which reinforced the fiber structure, and the elongation at break remained largely unchanged in contrast to non-fixed samples.

Fiber alignment also significantly influences mechanical behavior. In a study by Lee et al. [133], increasing the rotational speed of the collector during electrospinning led to improved fiber alignment, which enhanced the orthotropic mechanical properties of both PCL and PCL/SF composite fibers. For instance, adding 2 wt% SF increased the Young’s modulus to 27.47 MPa. However, higher SF content caused aggregation and reduced overall strength and flexibility, showing that both alignment and optimal composition are critical for tuning mechanical response.

Kumkun et al. [46] found that heating significantly enhanced the mechanical properties of SF/PVA scaffolds. Heated fibers exhibited a five-fold increase in tensile strength and a seven-fold improvement in elongation at break because of stronger molecular interactions between SF and PVA, leading to a more flexible polymer network. Overall, SF fibers demonstrate tunable mechanical properties through methanol treatment, fiber orientation control, and polymer blending, making them highly adaptable for biomedical applications such as tissue engineering and wound healing.

#### 4.2.6. PHBV and PHB

The family of polyhydroxyalkanoates (PHAs) has gained significant attention as biocompatible and biodegradable thermoplastic biopolymers. The two most studied polymers in the PHA group are poly(3-hydroxybutyrate) (PHB) and PHBV. Poly(3-hydroxybutyrate-co-3-hydroxyvalerate) (PHBV), a microbial polyester that is biodegradable, non-antigenic, and biocompatible, is a copolymer of polyhydroxybutyrate (PHB) and polyhydroxyvalerate (PHV) [46,136]. PHB is characterized by a high degree of crystallinity and rigidity with a high melting temperature. To enhance its chain flexibility, melting point, and processability, 3-hydroxyvalerate (HV) units are incorporated to create the biopolymer PHBV [137].

PHBV is an environmentally friendly biopolymer that microorganisms can naturally degrade in various environments, depending on the ratio of 3-hydroxybutyrate to 3-hydroxyvalerate. It has a wide range of applications, including in biomedicine, packaging, and agriculture. Because of its natural origin, biocompatibility, and biodegradability, PHBV is ideal for drug delivery systems, tissue engineering, and bioresorbable medical implants.

**Figure 11:**
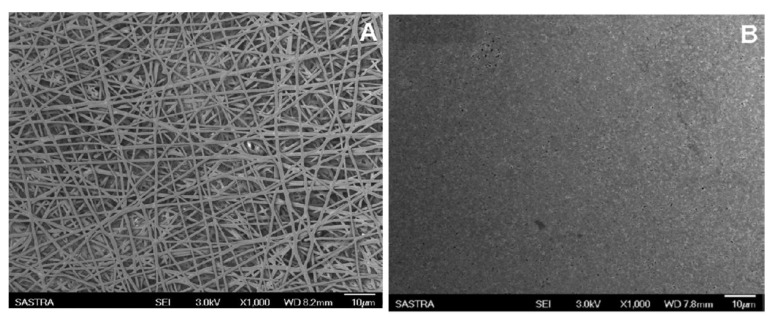
Scanning electron micrograph showing the surface morphology of (**A**) electrospun nonwoven PHBV fibers and (**B**) solvent-cast PHBV 2D film [138] (reprinted with permission).

The mechanical properties of PHBV fibers are influenced by solvent choice, polymer blending, fiber orientation, and processing methods. Kuppan et al. [138] compared electrospun PHBV fibers with solvent-cast 2D films for skin regeneration (Figure 11) and found that whereas the fibers had lower tensile strength (1.42 vs. 1.57 MPa) and Young’s modulus (1028 vs. 5619 MPa), they exhibited superior elasticity, higher porosity, and an accelerated degradation rate, making them more suitable for tissue engineering applications.

Polymer blending has shown significant improvements in the mechanics of PHBV fibers. A study comparing PHB, PHBV, and their blends in chloroform solution revealed that blending PHB with PHBV (75/25 *w*/*w*%) increased tensile strength from 1.8 to 2.45 MPa while also enhancing elongation at break. This improvement was attributed to better molecular chain entanglement and reduced brittleness of the polymer network [139].

Solvent choice and fiber orientation also influence mechanical behavior. Xin Lu et al. [136] demonstrated that electrospinning PHBV in 2,2,2-trifluoroethanol (TFE) instead of chloroform significantly improved tensile properties, increasing tensile strain from 3.73% to 25.54% and tensile stress from 1.17 to 1.88 MPa, because of finer fiber diameters (5200 to 690 nm) resulting from the varying solvent conductivity and volatility. Additionally, fiber alignment played a key role: aligned nanofibers exhibited a tensile stress of 8.86 MPa but lower strain (7.77%), whereas randomly oriented fibers showed significantly higher strain (71.49%) but reduced tensile stress (1.62 MPa).

Incorporating PHBV into PLLA fibers has been explored to enhance stiffness and flexibility. Increasing the PHBV content to 30% in PLLA fibers raised Young’s modulus from 41.5 to 73.5 MPa, attributed to stronger molecular interactions and enhanced crystallinity during electrospinning [140].

Alternative fiber fabrication methods, such as centrifugal spinning, have also been investigated. Upson et al. [141] produced PHBV fibers at 9000 rpm, achieving tensile strengths of 3 MPa and a Young’s modulus of 100 MPa, comparable to electrospun PHBV fibers, which typically exhibit strengths of 1.76 MPa and moduli of 125.7 MPa. Although centrifugal spinning resulted in slightly lower stiffness, it offered the advantage of higher production rates.

Nanoparticle incorporation has limited influence on the mechanics of PHBV fibers. Mayorga et al. [142] found that CuO nanoparticles, whether integrated via melt mixing or coating, did not significantly affect mechanical properties because of their low loading levels and uniform dispersion. Similar trends were observed with silver nanoparticles, where finely dispersed AgNPs had minimal impact on fiber strength and flexibility, suggesting that structural modifications may be a more effective strategy for enhancing PHBV fiber mechanics rather than adding nanofillers [137,143].

Therefore, PHBV fibers exhibit tunable mechanical properties through polymer blending, solvent selection, fiber orientation, and alternative processing methods. Although blending and controlled processing significantly enhance strength and elasticity, nanoparticle incorporation appears to have minimal effect, emphasizing the importance of optimizing polymer interactions and fiber morphology for improved mechanical performance.

Table 2 summarizes the mechanical behavior of electrospun fibers derived from natural biopolymers and their composites. It outlines techniques such as blending with synthetic polymers, reinforcement with nanofillers, and post-processing treatments aimed at improving strength, flexibility, or structural integrity.

**Figure 12:**
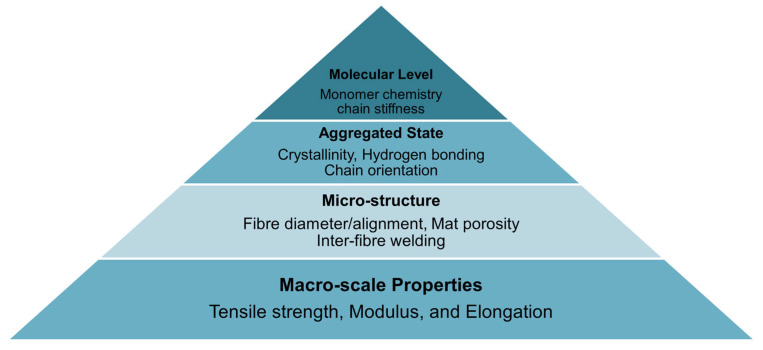
Structure–processing–property pyramid for biodegradable fibers.

To highlight the multiscale interactions that impact mechanical behavior in biodegradable fiber systems, a schematic structure–property pyramid (Figure 12) has been added. This diagram links molecular features (e.g., monomer chemistry, tacticity), aggregated-state attributes (e.g., crystallinity, hydrogen bonding), and microstructural factors (e.g., fiber alignment, mat porosity) to macroscale performance indicators such as tensile strength, modulus, and elongation [148]. It illustrates how processing strategies and material modifications at different scales collectively shape the final mechanical performance of fiber mats.

## 5. Future Perspectives

Biodegradable fibers and mats have a promising future as the demand for sustainable materials grows. Research will continue to focus on enhancing the strength, durability, and scalability of these fibers. A key challenge is improving the mechanical properties of natural and synthetic biodegradable polymers to replace traditional materials across various industries. New developments, such as combining different polymers, adding nanoparticles, and using specialized treatments, can enhance fiber strength and flexibility. Simultaneously, finding eco-friendly production methods will be essential to reduce waste and pollution. With further advancements, biodegradable fibers will find greater utility in medicine, packaging, and textiles, contributing to a more sustainable future. On the other hand, looking ahead, artificial intelligence and computational modeling offer exciting opportunities to predict and tailor mechanical behavior in biodegradable electrospun fibers. For instance, a recent study used machine learning models (including GAMLSS, decision trees, and neural networks) to predict fiber diameter, tensile strength, and modulus in gelatin-based fibers spun from green solvent blends, achieving significantly improved accuracy versus traditional regression approaches [149]. In another investigation, machine learning models (CART, SVM) accurately identified the structure–property relationships in crosslinked PVA electrospun scaffolds, reaching prediction R^2^ values of up to 0.93 for strength and modulus [150].

## 6. Conclusions

This paper reviews the mechanical properties of biodegradable fibers and mats on the basis of their production methods and whether they are synthetic or natural polymers. It highlights their potential as sustainable alternatives for various applications. The analysis elucidates that the intrinsic properties of biodegradable polymers such as PVA, PEO, PLA, PHBV, PCL, PGA, chitosan, collagen, starch, alginate, silk, and cellulose, alongside their processing techniques, significantly affect the mechanical strengths and limitations of the resulting fibrous structures. It was shown that fibers processed via electrospinning show promise because of their enhanced surface area and porosity, whereas melt-spinning and wet-spinning techniques are advantageous for industrial scalability and customizability. Including nanocomposites, chemical treatments, and fiber orientation strategies are key enhancement methods for tailoring mechanical properties such as tensile strength and elasticity. This review paves the way for future research to bridge the gap between material properties and application requirements. It aims to provide better insight for researchers in selecting the best fiber production methods, mechanical evaluation techniques, and appropriate polymers on the basis of their mechanical properties and applications. It also suggests several techniques for improving these properties. As the demand for bio-based and high-performance materials grows, the optimization of mechanical properties will play a critical role in expanding the applicability of biodegradable fibers.

## Figures and Tables

**Table 1:** Summary of polymer composition, fiber size, and mechanical properties of electrospun fibers based on synthetic polymers.

Main Polymer	Fiber Components	Average Fiber Diameter (nm)	Tensile Strength (MPa)	Elongation at Break (%)	Young’s Modulus (MPa)	Application and Processing Technique	Ref.
PVA	Isotropic PVA	292	4.21	-	64.7	Separation filters, wound dressing materials, tissue scaffold Electrospinning	[50]
	PVA/cellulose nanowhisker	-	7.84	-	132.8		
	aligned PVA	166	5.36	-	87.7		
	PVA/cellulose nanowhisker	149	10.5	-	190.5		
	PVA/cellulose nanowhisker	-	7.84	-	132.8		
	PVA/CS	155	3.65	-	-	Antibacterial activity Electrospinning	[42]
	PVA/CS/Ag P (0.25%)	148	3.78	-	-		
	PVA/CS/Ag NPs (0.5%)	144	4.52	-	-		
	PVA/CS/Ag NPs (1%)	139	3.74	-	-		
	PVA	328	7.31	186	29.2	Wound healing Electrospinning	[45]
	PVA/AgNPs	285	9.19	31	182.4		
	PVA–silk sericin (non-heated)	60–500	0.7	5	-	Biomedical applications (skin) Electrospinning	[46]
	PVA–silk sericin (heated)		3.7	35	-		
	PVA/γ-Fe_2_O_3_ porosity: 76.94%	301	60.31	-	114.53	Soft tissue engineering Scaffold Electrospinning	[53]
	Porosity: 80.68%	298	57.48	-	111.62		
	Porosity: 85.53%	260	54.93	-	108.80		
	Porosity: 90.85%	235	51.45	-	105.97		
	Porosity: 91.24%	220	50.28	-	105.27		
PEO	PEO	670–1620	1.8–4.23	445–517	4.88–15.3	General applications Centrifugal spinning	[17]
	CMCS-PEO	1.91–3.22 µm	0.8–2.2	11.2–22.8%	10.5–26.6	Wound dressing Centrifugal spinning	[58]
	Starch–PEO	2 µm	0.39–1.8	-	-	Drug delivery Centrifugal spinning	[59]
	PCL/PEO (75/25)	250–450	6.5	-	12–30	Surface Functionality	[57]
PGA And PLGA	PGA-PLA	900–1300	2.2–11	70–330	3.4–140	Biomedical applications Electrospinning	[63]
	PGA	300–1500	2.7	2.1	138	Tissue engineering Electrospinning	[64]
	PLGA (50/50)		2.8	2.2	144		
	PLGA (85/15)		1.9	1.95	114		
	PLGA (85/15)		1.9	1.95	114		
	PGA	480	-	3.5	1013.4	General application Electrospinning	[65]
	PGA (14 days)	-	-	1.5	2989		
	PGA	666	3.44	152	74	Tissue engineering (cell infiltration) Electrospinning	[56]
	PLGA	2211	5.9	276	141		
	PGA (sacrificial PEO)	609	3.32	171	66		
	PLGA (sacrificial PEO)	2294	6.04	144	86		
	PCL	1165	7.05	453	28		
	PCL (sacrificial PEO)	1171	2.57	488	9		
	PCL	245	1.02	32.43	7.75	Tissue engineering (blood vessel) Electrospinning	[66]
	PLGA	163	3.1	3.1	13.51		
	(PCL-PLGA) Coaxial	256	2.89	2.89	9.18		
PLA And PLLA	PLLA	850	2.7	261	1.15	Medical applications Electrospinning	[73]
	PLLA/TEAC	270	27.2	79	2.8		
	PLLA/formic acid	520	9.5	150	1.5		
	PLLA/TX-100	570	7.1	255	1.7		
	PLA	-	1.17	144	-	Bone tissue engineering Electrospinning	[78]
	PLA-OS		1.72	103	-		
	PLA	910	0.8	125	-	Wound healing Electrospinning	[79]
	PLA/Rosa/GO	720	2.6	50	-		
	PLA/PEG/RosA/GO	-	2.3	15	-		
	PLA	~360	1.5	-	-	Hard tissue engineering Electrospinning	[18]
	PLA/NCC	~280	3.3	-	-		
	PLLA	712	3.49	49	56.8	Cell adhesion and growth Electrospinning	[107]
	PLLA/PLA-Lig10	485	3.25	21	55.2		
	PLLA/PLA-Lig20	525	2.49	19	56.1		
	PLLA/PLA-Lig30	480	2.55	20	54.3		
	PLLA/PLA-Lig40	417	2.58	16	66		
	PLLA/PLA-Lig50	350	2.56	11	66.8		
	PLA (CF/DMF (80:20 *v*/*v*))	-	5.8	29.8	305	Biomedical applications Electrospinning	[98]
	PCL	-	1.6	82.1	7.5		
	PLA-PCL (70/30 *w*/*w*)	-	2.5	155	67.5		
	PLLA (treated at 120 C)	796	5.6	56.9	165	Tissue engineering Electrospinning	[74]
	PLLA (DCP crosslinkers)	895	8.2	59.4	203		
	PLLA (DCP-TAIC crosslinkers)	-	8.78	47.3	208		
	PLLA	323	3.8	76.1	91.7		
	PLLA	323	3.8	76.1	91.7	Tissue regeneration (human annulus fibrosus tissue) Electrospinning	[82]
	(PLLA) After 180 days in PBS	330	5.2	38.9	136.4		
PCL	PCL	219	2.9	190	16.3		
	(PCL)	214	3.5	81.4	21.6		
	PLLA-PCL	720	4.1	148.3	71.6		
	PLLA-PCL After 180 days in PBS	671	6.2	87.3	125.6		
	PLA	2500–7500	0.18	-	-	Cell culture (melt-blown)	[83]
	PLA	20,000	3.77 cN/dtex	18	80.43 cN/dtex	Packaging Melt spinning	[86,87]
	PLA, 350 rpm	9980	1.51	7.77	26.18	Tissue engineering Centrifugal spanning	[84]
	PLA, 900 rpm	8270	2.34	8.47	46.21		
	PLA, 1500 rpm	5990	3.31	7.47	66.77		
	PLA (CF/DMF (80:20 *v*/*v*))	-	5.8	29.8	305	Biomedical application Electrospinning	[98]
	PCL	-	1.6	82.1	7.5		
	PLA-PCL (70/30 *w*/*w*)	-	2.5	155	67.5		
	PCL	1830	4.1	222	48	Drug delivery Electrospinning	[101]
	PCL (E-beam treatment)	1870	1.3	175	5		
	PCL	721	11.53	-	8.41	General Electrospinning	[102]
	PCL (DCM vapor treatment)	693	21.4	-	16.5		
	PCL/Gel	-	1.2	28	-	Medical application Electrospinning	[104]
	PCL/Gel/HNTs	-	2.3	67	-		
	PCL	824	3.14	-	40.76	Wound dressing Centrifugal spinning	[106]
	PCL/Gel (70/30)	534	2.91	-	84.87		
	PCL/Gel (50/50)	367	1.7	-	82.70		
	PCL/Gel (30/70)	265	1.22	-	72.76		
	PCL fiber (AFM)	440–1040	12	98	62	Biomedical devices Electrospinning	[36]
	PCL fiber (AFM)	-	-	-	4	Tissue engineering Scaffolds Electrospinning	[105]
	PCL fiber/HAp (AFM)	-	-	-	20		
	PCL fiber (AFM)	20–25	257–340	127–133	500–3000	General application Electrospinning	[93]

**Table 2:** Summary of polymer composition, fiber size, and mechanical properties of electrospun fibers based on natural polymers.

Main Polymer	Fiber Components	Average Fiber Diameter (nm)	Tensile Strength (MPa)	Elongation at Break (%)	Young’s Modulus (MPa)	Application and Processing Technique	Ref.
Chitosan	CS (1% acetic acid)	325	0.7	19	3.6	Tissue engineering Electrospinning	[109]
	CS (2% acetic acid)	350	1.2	17	7		
	CS (6% acetic acid)	450	0.3	12	7		
	Chitosan (10–30%)–PDO	0.26–0.31 µm	4.6–6.3	-	14–68 MPa	Vascular tissue engineering Electrospinning	[112]
	CS–pullulan	-	11.36	4.53	344.3	Active packaging Electrospinning	[113]
	CS–pullulan–crosslinker (cinnamaldehyde)	-	22	2.83	1150.9		
	CS-PVA	155	3.65	-	-	Biomedical application Electrospinning	[51]
	CS-PVA-AgNPs (0.25%)	148	3.78	-	-		
	CS-PVA-AgNPs (0.5%)	144	4.52	--			
Collagen	Collagen (AFM)	60–80 µm	650	-	3.2 GPa	Tissue engineering Electrospinning	[119]
	Collagen–cathepsin K (AFM)		241	39	1.9 Gpa		
	Collagen–PCL 9:1	318	4.2	4.74	-	Wound-healing applications Electrospinning	[118]
	Collagen–PCL–crosslinker (5/5)	388	7.65	8.93	-	Nerve tissue engineering Electrospinning	[144]
	Collagen–PCL–crosslinker (9/1)	480	12.61	11.76	-	Tissue engineering Electrospinning	[145]
Cellulose	PLA/nanocrystalline cellulose (NCC) (0–2%)	280–320	1.5–3.3	-	-	Tissue engineering Electrospinning	[18]
	PLA/nanocrystalline cellulose (NCC)	600–700	0.5–2.1	-	-	Tissue engineering Electrospinning	[121]
	PCL–cellulose nanocrystals (CNCs) (0–20%)	350–600	2.7–7.2	110–180%	-	General application Electrospinning	[123]
	PLA/nanocrystalline cellulose (NCC) 5%	642	6.3	70	125.6	Tissue engineering scaffold Electrospinning	[122]
	PCL–cellulose nanocrystals (CNCs) 65–95% (AFM)	11.2 µm	705	-	4.9 GPa	Tissue engineering Melt spinning	[124]
	(PLA-PCL)-BC 70/30	5–18.5 µm	9.05	-	19.61	Wound healing Centrifugal spinning	[125]
Starch	Starch (in formic acid solution)	80–300	9.38	26	264	General application Electrospinning	[130]
	Starch–PCL	156–476 µm	13	-	20	Tissue engineering Electrospinning	[109]
	Starch–PLA (50)	-	27	-	33		
	Starch–PLA (70)	-	40	-	162		
	Starch–EVA	-	25	-	85		
	Starch–PVA	247–301 µm	0.88–11.54	-	-	Wound dressings Wet extrusion	[128]
SF	SF (10%) SF after treatment with methanol	85–206	16.03 25.41	21.7 11.9	253 MPa 490 MPa	Air permeability Electrospinning	[135]
	SF (20%) (central angle 60°)	230	4.4	7.99	30	Tissue engineering Electrospinning	[134]
	SF after treatment with methanol		15.3	12.3	150		
	2 wt% PCL/SF	-	1.3	28.27	27.67	Biomedical application Electrospinning	[133]
	PCL	-	1.3	70.47	14.06		
	PCL/SF (70/30)	539	4.7	62.7	12.73	Tissue engineering Electrospinning	[146]
	PCL/SF	242–618	11.2		70	Wound healing Electrospinning	[147]
	PLA	10,365	1.14	15.97	70.21	Tissue engineering scaffold Electrospinning	[131]
	PLA75/SF25	665	0.47	26.22	20.51		
	PLA50/SF50	693	1.05	17.51	16.49		
	SF/PVA	60–500	0.7	5	-	Biomedical application Electrospinning	[46]
	SF/PVA (thermal treated)	-	3.7	35	-		
PHBV	PHBV fiber	724	1.4	-	1028	Skin tissue engineering Electrospinning	[138]
	PHBV 2D film	-	1.57	-	5619		
	PHBV	105 µm	1.6	-	127	Antibacterial fibers Electrospinning	[139]
	PHB	383–5200	1.8	-	147	Bone tissue engineering Electrospinning	[136]
	PHBV-PHB (25/75)		2.45		140		
	PHBV (chloroform solution)		1.17	3.73			
	PHBV (TFE solution)		1.88	25.54			
	PHBV-PLA (30/70)	2890			73.5	Bone regeneration Electrospinning	[140]
	PLA		-	-	41.5		
	PHBV	0.5–3 μm	3	-	100	Biomedical application Centrifugal methods	[141]
	PHBV	-	1.76	-	125.7	Tissue engineering	[136]
